# Large Splenic Cyst: A Rare Presentation

**DOI:** 10.7759/cureus.14435

**Published:** 2021-04-12

**Authors:** Amrit Pal Singh Rana, Sudhir Khichy, Harkanwalpreet Kaur, Harinder Singh, Parampreet Singh Sandhu

**Affiliations:** 1 Surgery, Baba Farid University of Health Sciences, Faridkot, Faridkot, IND; 2 Surgery, Guru Gobind Singh Medical College, Baba Farid University of Health Sciences, Faridkot, Faridkot, IND

**Keywords:** nonparastic cyst, epithelial cyst, spleen

## Abstract

Cystic lesions of the spleen are rare lesions and can be parasitic or nonparasitic. Nonparasitic cysts are of two types: primary cysts and secondary pseudocysts. Primary cysts of the spleen are very rare and are also called true, congenital, epidermoid, or epithelial cysts. Splenic cysts are usually asymptomatic and often found incidentally during imaging studies. We are presenting a case of a 19-year-old female with a large splenic cyst which was surgically treated by open splenectomy.

## Introduction

Cystic lesions of the spleen are infrequently seen in routine surgical practice and have been classified according to the pathogenesis of the cyst [1]. Nonparasitic splenic cysts are classified as congenital, neoplastic, traumatic, or degenerative. Parasitic cysts are caused mainly by *Echinococcus granulosus *infestation [2]. Nonparasitic cysts are of two types: true cysts (with epithelial lining) and pseudocysts (without epithelial lining) [3]. True splenic cysts are predominantly seen in paediatric and adolescent age groups and constitute 10% of all nonparasitic cysts of the spleen [4]. We present here a rare case of a large splenic cyst that adhered to the left lobe of the liver and diaphragm and was surgically treated by open total splenectomy.

## Case presentation

**Figure 1:**
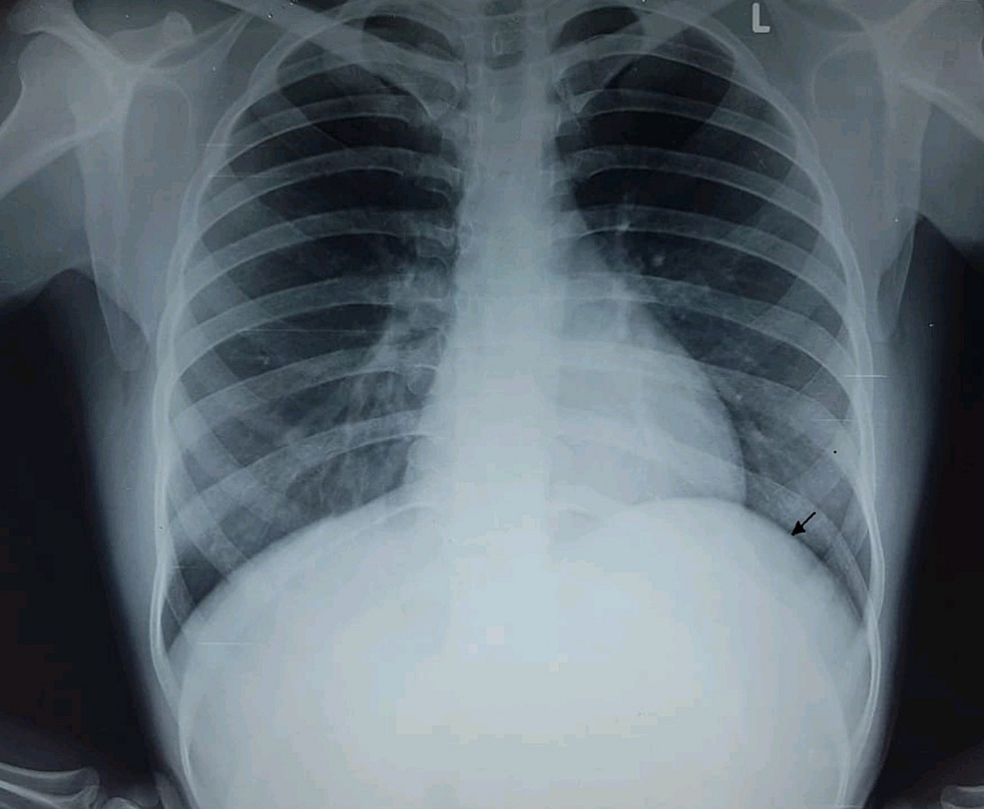
X-ray chest (PA view) depicting raised left hemidiaphragm due to a large cystic lesion left hypochondrium (arrow) PA: posteroanterior

A 19-year-old female immigrant worker presented to the surgical outpatient department with complaints of dull upper abdominal pain of several days duration. There was a history of an increase in pain after food intake, not relieved on medications like antacids and pain killers. There was no complaint of diarrhoea or constipation or fever. She had no other traumatic event in the abdomen. Her medical history was notable only for appendectomy performed several years ago. On physical examination, vital signs were stable and all laboratory findings were within the normal range. Abdominal examination revealed a soft, smooth non-tender mass palpable in the left-upper quadrant. Ultrasonography abdomen showed a large cystic mass of the spleen with a query of splenic abscess. On X-ray chest, the left dome of the diaphragm was seen raised (Figure 1).

**Figure 2:**
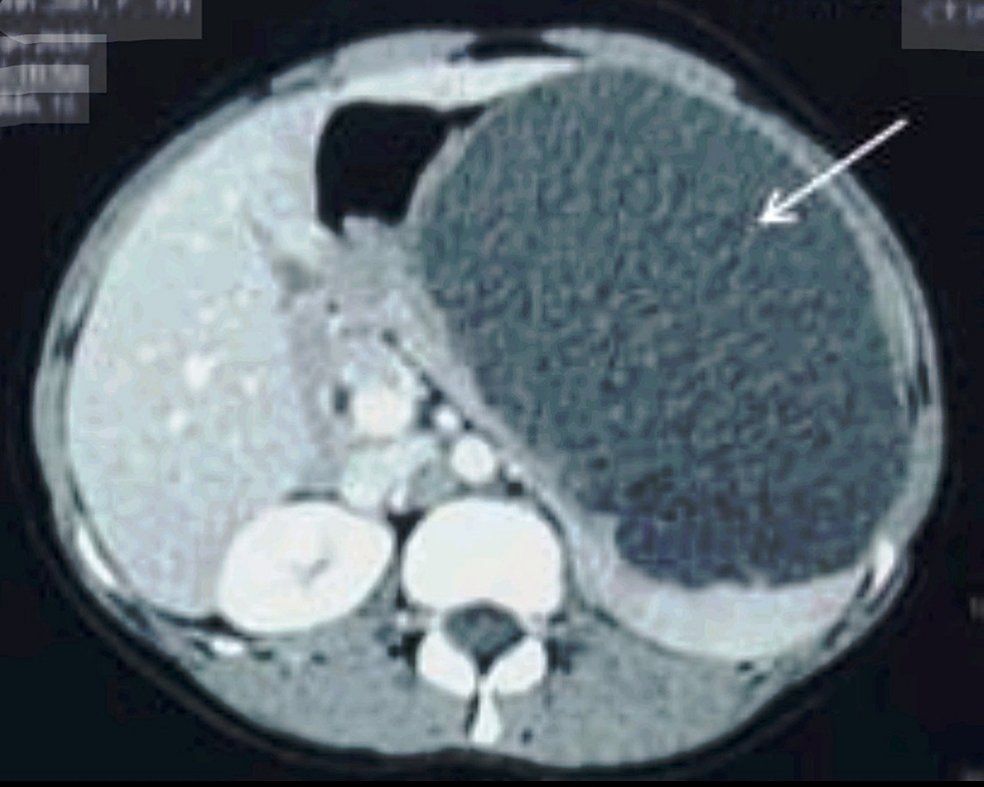
CT abdomen showing a large cystic lesion ( white arrow ) of spleen

The possibility of hydatid cyst kept was ruled out by negative hydatid serology. Computed tomography (CT) of the abdomen was conducted after administering oral and intravenous contrast. Axial sections showed a huge cystic lesion of the spleen filled with fluid, compressing the stomach and displacing the left lobe of the liver and left diaphragm upwards, which measured 15 x 12 x 10 cm in size (Figure 2). No intracystic septa or air pockets were seen.

**Figure 3:**
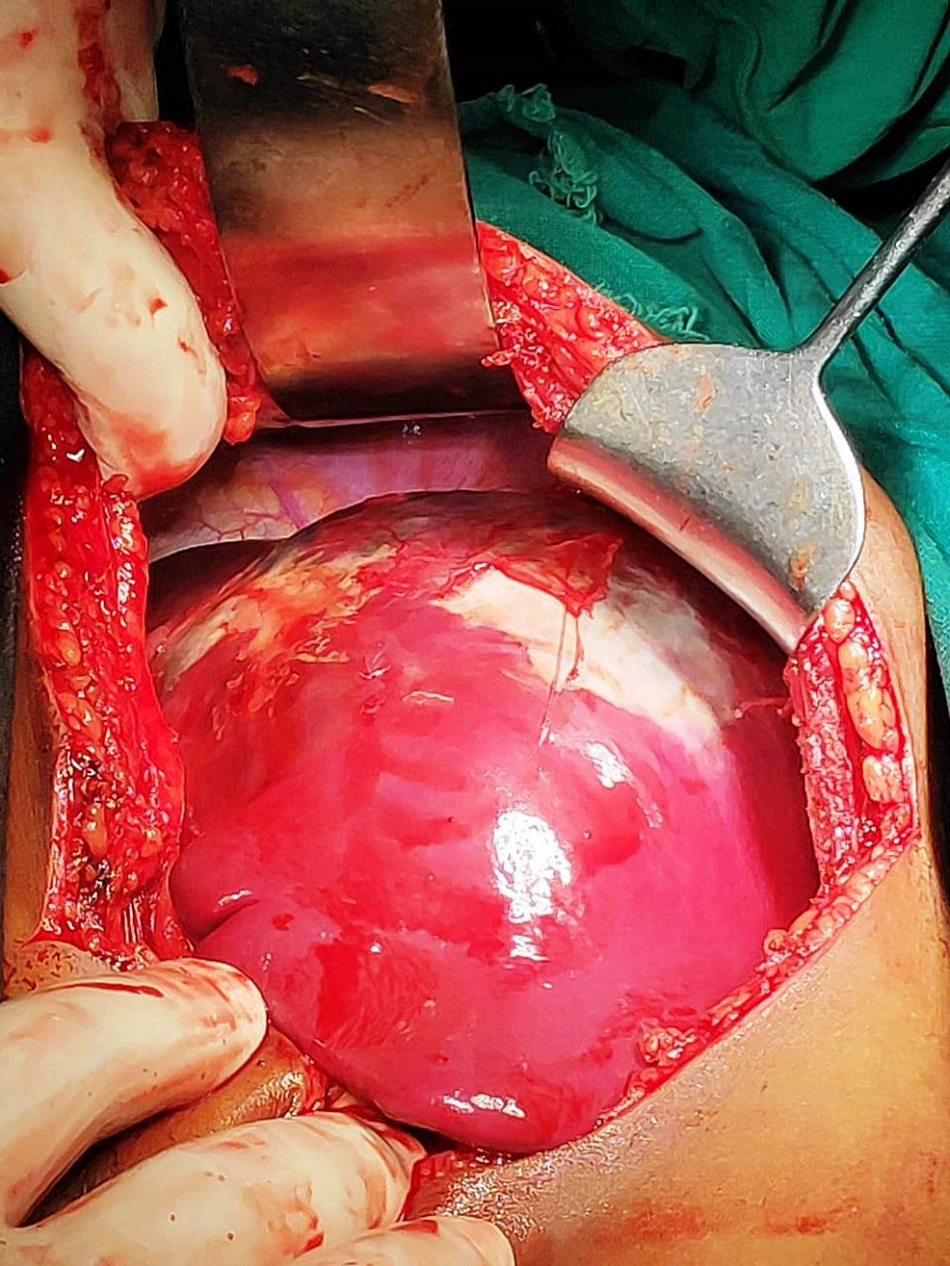
Intraoperative view of large cyst of spleen

**Figure 4:**
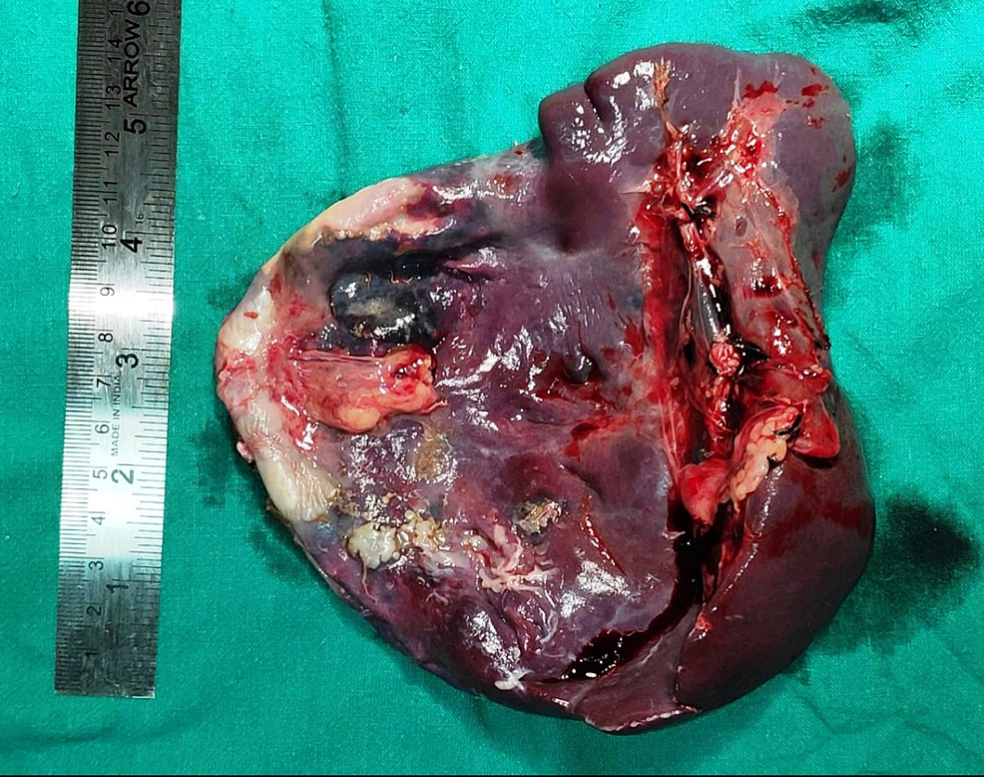
Gross view of specimen large cystic lesion of the spleen

Total splenectomy was planned, intraoperatively the huge splenic cyst was found occupying more than the upper two-thirds of the spleen involving the hilum (Figures 3, 4). The cyst adhered to the left lateral side of the liver and the left side of the diaphragm.

**Figure 5:**
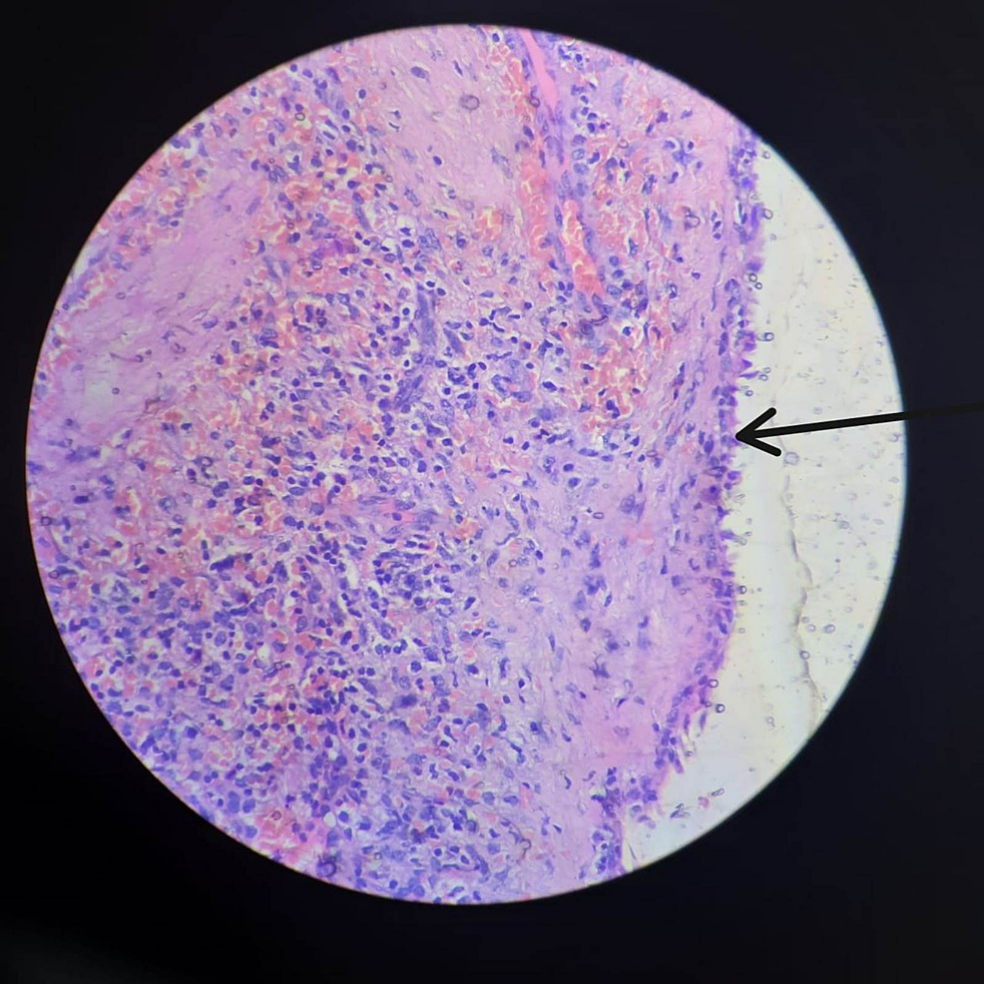
Showing epithelial lining (arrow), haematoxylin and eosin (40x)

About 1300 ml of straw colored cystic fluid was carefully aspirated to avoid rupture into the operation field, followed by the total splenectomy. A drain was left near the splenectomy site. The cystic fluid cytology showed foamy macrophages only and histopathology revealed epithelial splenic cyst (Figure 5).

The patient was vaccinated postoperatively and was discharged on the seventh postoperative day without complications.

## Discussion

Splenic cysts are categorized based on the presence or absence of an epithelial lining, etiology, and pathogenesis. On the basis of the causative agent, splenic cysts are divided into two types: parasitic cysts and non-parasitic cysts [5, 6]. Parasitic cysts are usually seen in endemic areas [7]. The histogenesis of primary cyst is thought to develop from mesonephric tissue in the developing spleen in early embryonic life. The suggested hypothesis is the entrapment of mesothelial cells of the peritoneum in the splenic parenchyma during embryogenesis in the intrauterine life. Another theory suggests the origin of cysts from the normal lymph spaces in the spleen. These cysts are more common in children and adolescents with slight female preponderance [8, 9]. The most common presentation (30-40%) is painless mass in the left hypochondriac region with pain due to pressure symptoms. In our case also, the patient was a 19-year-old female presenting with belching pain in the abdomen [10]. Although ultrasonography may show septations in the cyst or irregular walls, and computed tomography or magnetic resonance imaging can help in identifying the morphology and location of the cyst in the spleen, nature of the fluid, and relation with adjacent structures, final diagnosis depends upon histopathological diagnosis [11]. Treatment of the cyst depends upon the size of the cyst and related symptoms. Asymptomatic cysts that are <5 cm in diameter, particularly non-parasitic ones, are best followed conservatively [12]. And for spontaneous resolving of small cysts, serial imaging is opted for as the treatment of choice. Due to vulnerability for haemorrhage, rupture, and infection, larger cysts >5 cm in diameter and symptomatic cysts must be treated surgically [13]. In case hilum is involved, splenectomy remains a relatively safe procedure [14]. A large intrasplenic cystic lesion of the spleen involving the hilum can be adherent to supplying vessels and thus should be treated by splenectomy - either open or laparoscopic procedure - and a conservative approach should be avoided [15]. One of the authors has also reported a case of a large splenic epidermoid cyst that was removed by open total splenectomy [16].

## Conclusions

Although the diagnosis of splenic cyst is made by imaging preoperatively, histopathological examination is required to ascertain the type, whether the cyst is true or false. Total splenectomy - either by open or laparoscopic technique - is the treatment of choice, especially if the cyst is large and is extending into the hilum, to avoid recurrence and complications.
